# Advancing Pregnancy and Pediatric Science through the NIH Transporter Elucidation Network

**DOI:** 10.1002/cpt.70517

**Published:** 2026-10-15

**Authors:** Joanne Wang, Jashvant D Unadkat, Samuel LM Arnold, John K Fallon, Pouria Fattahi, Dan Dongeun Huh, Nicholas Ieronimakis, Edward J Kelly, Svetlana Lutsenko, Robert M Stroud, Jacqueline B Tiley, Jia Yang, Xujia Zhou, Hao Zhu, Rajini Rao, Kathleen M Giacomini, Lauren M Aleksunes

**Affiliations:** ^1^ Department of Pharmaceutics School of Pharmacy University of Washington Seattle Washington USA; ^2^ Division of Pharmacoengineering and Molecular Pharmaceutics, and Center for Nanotechnology in Drug Delivery UNC Eshelman School of Pharmacy, University of North Carolina at Chapel Hill Chapel Hill North Carolina USA; ^3^ Department of Bioengineering School of Engineering and Applied Science, University of Pennsylvania Philadelphia Pennsylvania USA; ^4^ Regenerative Bioscience Center, School of Chemical, Materials and Biomedical Engineering University of Georgia Athens Georgia USA; ^5^ Division of Clinical Investigation Madigan Army Medical Center Tacoma Washington USA; ^6^ Department of Physiology, Pharmacology and Therapeutics The Johns Hopkins University School of Medicine Baltimore Maryland USA; ^7^ Department of Biochemistry and Biophysics University of California San Francisco California USA; ^8^ Division of Pharmacotherapy and Experimental Therapeutics, UNC Eshelman School of Pharmacy University of North Carolina at Chapel Hill Chapel Hill North Carolina USA; ^9^ Department of Bioengineering and Therapeutic Sciences University of California San Francisco California USA; ^10^ Center for Biomedical Informatics and Genomics, School of Medicine Tulane University New Orleans Louisiana USA; ^11^ Department of Pharmacology and Toxicology Ernest Mario School of Pharmacy, Rutgers University Piscataway New Jersey USA

## Abstract

Human gestation is characterized by dynamic changes in maternal physiology and maturation of tissue functions before and after birth. The fetus and neonate are highly dependent upon transfer of maternal nutrients via the placenta and lactating mammary gland, which frequently involve selective membrane transporter proteins. At the same time, developmental changes in fetal and infant transport mechanisms critical for regulating absorption at the intestinal and blood–brain barriers are occurring. Coordinate regulation of cellular entry and efflux of nutrients, supplements, and xenobiotics, including drugs and environmental chemicals, must occur during this critical developmental period. In 2023, the National Institutes of Health launched the Transporter Elucidation Network (TEN) across four centers. This initiative applies experimental, computational, and translational approaches to (1) identify SoLute Carrier (SLC) superfamily and ATP‐binding cassette (ABC) transporters in the human placenta, mammary gland, as well as the pediatric brain and intestinal barriers and, (2) characterize their localization, expression, function, and regulation. The Network seeks to identify functional determinants of transporters as gatekeepers of entry to placenta, mammary gland and brain, and applies structural biology, proteomics, metabolomics, organoids, liquid biopsies, and microphysiological systems to this end. Computational strategies, such as machine learning, virtual screening, physiologically based pharmacokinetics, and perinatal epidemiology complement our experimental approaches. This paper highlights the TEN's multidisciplinary strategies to address current challenges in perinatal and pediatric transporter research and its commitment to advancing state‐of‐the‐art tools and new approach methodologies to fill critical gaps in our understanding of nutrient and xenobiotic exposure during early human development.

## BASICS OF SLC AND ABC TRANSPORTERS

Due to their transport functions and strategic localization at tissue barriers, membrane transporters play essential roles in the absorption, distribution, and elimination of nutrients and xenobiotics.^1^, ^2^ Most transporters can be classified into two major superfamilies: the ATP‐binding cassette (ABC) transporters and the solute carrier (SLC) transporters. ABC transporters utilize ATP hydrolysis to drive vectorial transport and, in many cases, to actively efflux substrates out of cells. In humans, there are 48 ABC transporters grouped into 7 families. Additional superfamilies of ATP‐driven transporters include the P‐type ATPases, V‐type or vacuolar ATPases and F‐type or ATP synthases. Whereas the V and F‐type ATPases exclusively transport protons in mammalian cells, the P‐type ATPases have a broad specificity including cations (Na^+^, K^+^, Ca^2+^, Cu^2+^), peptides, and lipids. The SLC superfamily is considerably larger, comprising over 450 members organized into more than 65 families. SLC proteins, which do not rely directly upon ATP hydrolysis to drive transport, exhibit diverse transport modes including facilitated transport, ion‐coupled active transport, or antiport/exchange mechanisms. Many ABC and SLC transporters are located on the plasma membrane, and in polarized tissue barriers they are often asymmetrically distributed with predominant expression on either the apical or basolateral membrane, working together to mediate the transepithelial movement of solutes. Additionally, some transporters are localized to intracellular membranes, facilitating solute exchange in organelles.

Transporters play a critical role in determining the disposition of nutrients and xenobiotics in the fetus, neonates, and infants.^3^, ^4^, ^5^, ^6^ For example, SLC transporters in the placenta transfer essential nutrients and hormones, thereby supporting and regulating fetal growth and development (**Figure** **1** **a**).^5^ Conversely, efflux transporters at the placental barrier restrict the entry of xenobiotics, protecting the fetus from potentially harmful substances circulating in the maternal blood.^4^, ^10^ During lactation, transporters in mammary epithelial cells regulate the concentrations of nutrients, vitamins, and minerals in breast milk (**Figure** **1** **b**).^11^, ^12^ Specific transporters in both superfamilies can facilitate the active transfer of drugs into human breast milk, potentially increasing the risk of infant exposure to drug‐induced toxicity.^11^ As barrier functions in neonates and infants continue to mature, transporters in tissues such as the developing gastrointestinal tract (Figure 1c) and blood–brain barrier (BBB) (**Figure** **1** **d**) become increasingly important for nutrient uptake as well as protection against xenobiotics. Further mutations in these transporters may have important implications for fetal and neonatal health.

**Figure 1:**
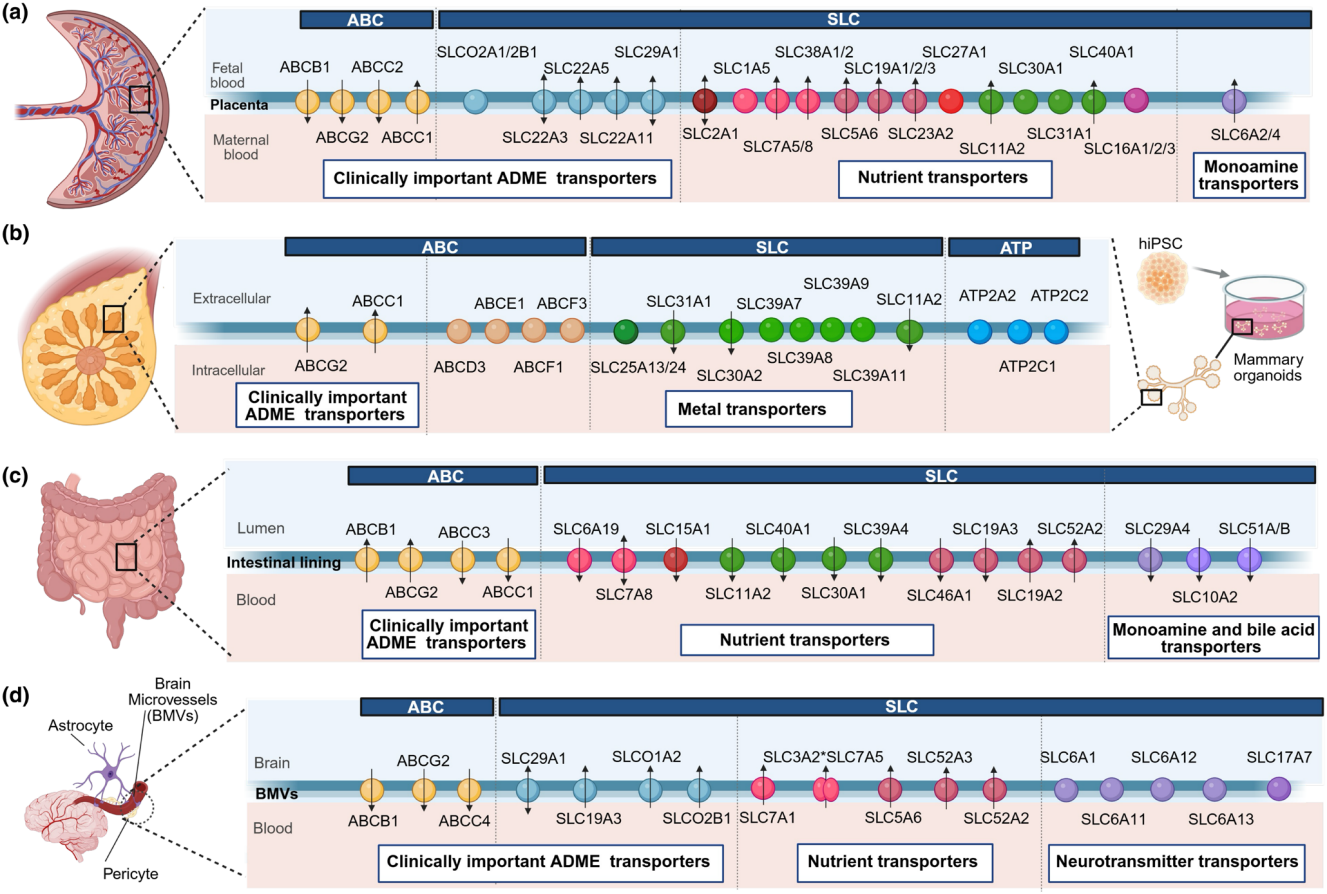
Proteomic detection of perinatal and pediatric transporters. Schematic representation of selected ABC and SLC transporters identified in human placenta (**a**), mammary gland/organoids (**b**), intestine (**c**), and brain microvessels (**d**). Transporters were included based on evidence of relevance to drug, nutrient, and chemical disposition. Arrows indicate the predominant net direction of transport based on literature; membrane localization is simplified where evidence remains incomplete. Color legend: Yellow = ABC transporters; Light blue = Clinically important ADME SLC transporters; Shades of red and magenta = Nutrient transporters with micronutrient transporter such as SLC19A1 colored as dark magenta; Green = metal transporters; Purple = monoamine, glutamate, GABA and bile acid transporters; Dark blue = ATPases. References include^7^, ^8^, ^9^ and unpublished data.

## PHYSIOLOGICAL CHANGES IN PREGNANCY AND EARLY DEVELOPMENT

The period following week 13 of pregnancy is typically referred to as the fetal period, with parturition denoting the start of the neonatal period. The perinatal period describes the time frame around birth and typically starts at week 22 through 7 days after birth^13^ though some researchers extend this description to include the entire first year of birth.^14^ This is a dynamic period of tissue growth and functional development.

Pregnancy produces a host of gestational age‐dependent physiological changes in the maternal‐fetal (m‐f) compartments.^15^, ^16^ For example, maternal cardiac output increases, resulting in changes in blood flow to various organs, more body water, decreased plasma protein concentrations, and higher concentrations of certain hormones such as cortisol, progesterone, and estrogens.^17^ Included in these physiological changes is a change in activity (and likely expression) of transporters and metabolizing enzymes. Accumulating evidence suggests that many renal transporters are induced during human pregnancy^18^, ^19^ while some hepatic transporters are repressed.^20^ In the placenta, transporters can limit or facilitate fetal exposure to nutrients and xenobiotics taken by the mother. For example, P‐glycoprotein (P‐gp/*ABCB1*) and Breast Cancer Resistance Protein (BCRP/*ABCG2*) localize to the apical membrane of the syncytiotrophoblasts, placenta cells that form a multinucleated barrier in contact with maternal blood (**Figure** **1** **a**). These transporters prevent entry of many xenobiotics into the fetal compartment. Their concentration per mg of placenta decreases with gestational age, but their total abundance is maintained by the continued growth of the placenta.^21^

The ontogeny of transporters in the offspring, including the intestine and BBB, is even less studied. In the pediatric liver, the abundance of organic cation transporter (OCT) 1/*SLC22A1*, organic anion transporting polypeptide (OATP/*SLCO*) 1B3, and P‐gp increases to ages 12 to 16 years when it reaches values that are comparable to those seen in adults.^22^, ^23^ Similarly, in neonates, the renal abundance of P‐gp, organic anion transporter (OAT) 1/*SLC22A6*, OAT3/*SLC22A8*, and OCT2/*SLC22A2* increases with postnatal age.^7^ The ontogeny of abundance and activity of transporters in the intestine or BBB is under active investigation. Such quantification will facilitate prediction of bioavailability and tissue distribution of nutrients and xenobiotics.

## ESTABLISHMENT OF THE TRANSPORTER ELUCIDATION NETWORK (TEN)

Recognizing the scarcity of data about human transporters during development, the *Eunice Kennedy Shriver* National Institute of Child Health and Human Development (NICHD) created the Pediatric Transporter Working Group in 2013. In their white paper,^24^ the working group identified the need for research into transporter ontogeny in humans, providing a starting point for the NICHD, the National Institute of Drug Abuse, and the Office of Dietary Supplements to establish the TEN. Through the creation of four Transporter Elucidation Centers (TECs) in 2023, the TEN was designed to: (1) elucidate and validate human models of transport across the placenta (**Figure** **1** **a**), the lactating mammary gland (**Figure** **1** **b**), and the developing gut (**Figure** **1** **c**) and BBB (**Figure** **1** **d**), (2) identify the substrates and transport properties of understudied SLC and ABC transporter genes and genetic variants, and (3) enhance the pipeline of investigators that study transporter biology. The TEN broadly aims to capture the entire period of pregnancy through 18 years of age in their research. Collectively, the TEN has six priority goals to achieve this mission (**Table** **1**). Details of the four TECs selected by NIH through competitive grant submissions are provided. These proposals included single institution as well as multi‐institution centers. In this section, we provide an overview of the 4 Centers and briefly highlight their experimental approaches which are described in more detail later in this commentary.

**Table 1:** Goals of the NIH Transporter Elucidation Network, which Focuses on Transporters in the Human Placenta, Mammary Gland, Intestine, and Blood Brain Barrier^a^

Methodologies	Disseminate new methods and tools to study transporters
	Test novel platforms to identify functional roles of transporters
Science	Establish novel xenobiotic/nutrient transporter interactions
	Identify functions of poorly characterized SLC/ABC transporters
Dissemination	Collaborate on joint publications between centers
	Create repositories of relevant transporter datasets

^a^ The TEN has six core goals that centered on novel transporter methodologies and experimental approaches, scientific inquiry, and dissemination of resources, datasets, and research findings.

### InTEC

The Integrated Transporter Elucidation Center (InTEC) spans five institutions, including Rutgers University, Tulane University, the University of Pennsylvania, the University of North Carolina at Chapel Hill, and the University of Rochester Medical Center. This team brings together expertise in perinatal pharmacology and toxicology, biomedical engineering, computational biology, machine learning, proteomics, epidemiology, and quantitative systems pharmacology. InTEC is (1) defining and identifying the transporter proteome in placentas using maternal extracellular vesicles, case–control studies, and longitudinal birth cohorts, (2) predicting and testing novel substrates, inhibitors, and regulators of transporters using cheminformatics, machine learning (ML), metabolomics, and screening assays, and (3) evaluating and advancing New Approach Methodologies (NAMs) for studying transporters, such as the glucose transporter 1 (GLUT1/*SLC2A1*), in a perfusable vascularized placental model (**Figure** **2**). Key areas of focus include the identification of novel transporter functions in human placentas as well as characterizing how disease, nutritional supplements, drugs, and environmental chemicals impact placental transporters.

**Figure 2:**
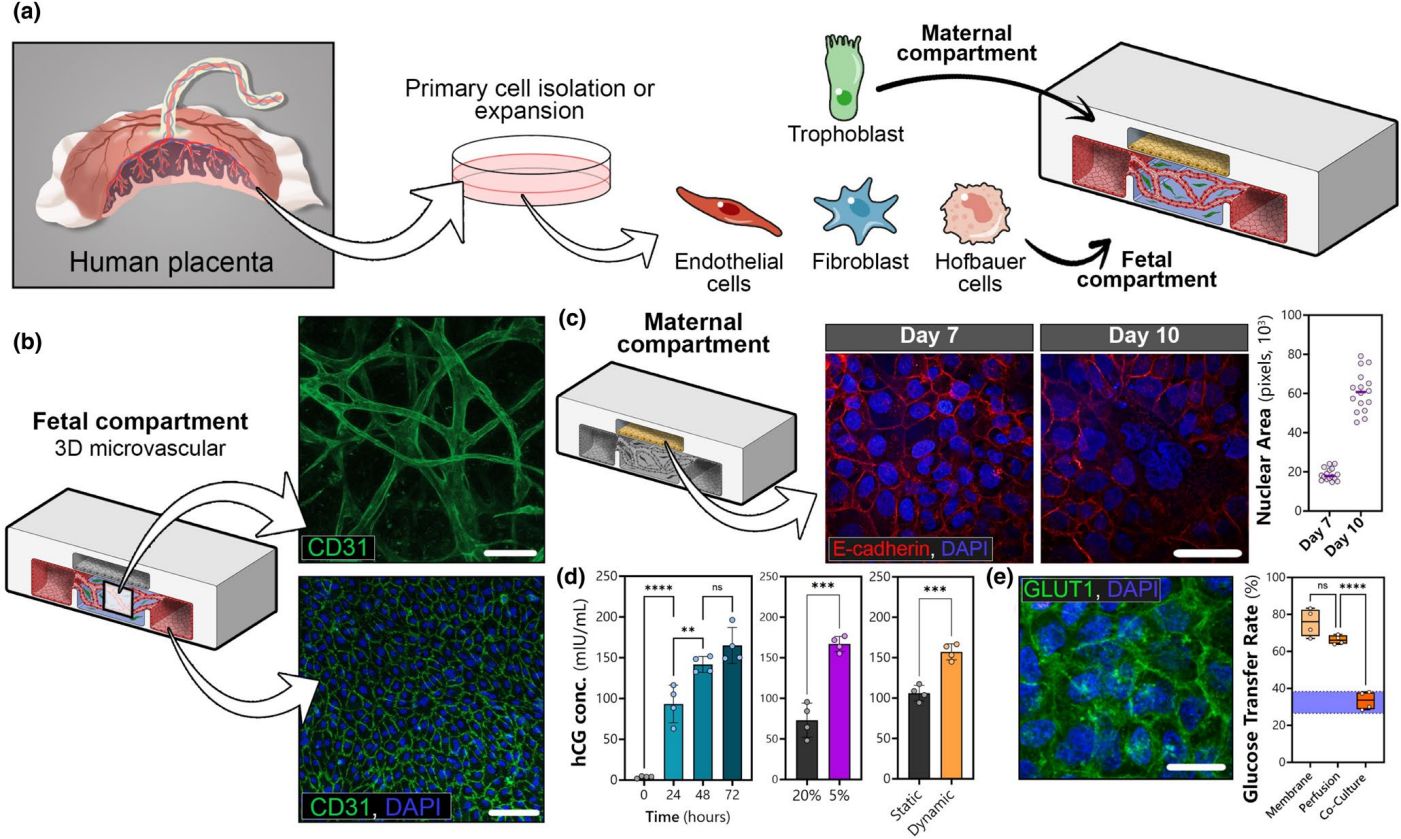
Modeling vascularized placenta‐on‐a‐chip microfluidic system. (**a**) Schematic representation of the human placenta highlighting the placental barrier and the workflow for primary cell sourcing. Primary placenta cells, including trophoblasts, endothelial cells, fibroblasts, and Hofbauer cells, were incorporated into the device to reconstruct maternal and fetal compartments. (**b**) Endothelial cells (CD31^+^) and fibroblasts self‐organize into microvascular networks in a gel channel over 7 days. Confocal projections of the middle and side channels are shown. (**c**) Trophoblast maturation in the maternal compartment. E‐cadherin staining shows epithelial organization at days 7 and 10, with nuclear area quantification indicating progressive syncytialization over time. (**d**) β‐hCG hormone secretion is increased over 72 hours and is further enhanced under reduced oxygen tension and dynamic flow compared with static culture. (**e**) GLUT1 transporter expression confirms nutrient transport capacity, and glucose transfer measurements show increased transport under perfusion and co‐culture conditions relative to membrane controls.

### UWTEC

Based at the University of Washington (UW) School of Pharmacy, with collaborators at the Madigan Army Medical Center, the mission of the UWTEC is to identify, quantify, and functionally characterize SLC and ABC transporters in the developing human placenta and the small intestine to better understand nutrient uptake and drug exposure in the fetus, neonates, and infants. The collaborative team brings together complementary expertise in transporter biology and perinatal pharmacology, integrating state‐of‐the‐art global and targeted proteomics and transcriptomics, human intestinal organoids, *ex vivo* human placenta perfusion, and physiologically based pharmacokinetic (PBPK) modeling and simulation. UWTEC is defining transporter abundance and ontogeny in pediatric intestines and human placentas. This includes the propagation of organoids from postnatal human intestines capable of differentiation and culturing on Transwells™ and in 3D suspension (**Figure** **3**). Complementary *in vitro* and *ex vivo* models are being used to identify novel substrates and define the cellular localization and activity of highly abundant transporters in these tissues. This research will reveal the mechanisms governing chemical disposition during early human development, which can be leveraged to predict the efficacy and toxicity of xenobiotics and nutrients.

**Figure 3:**
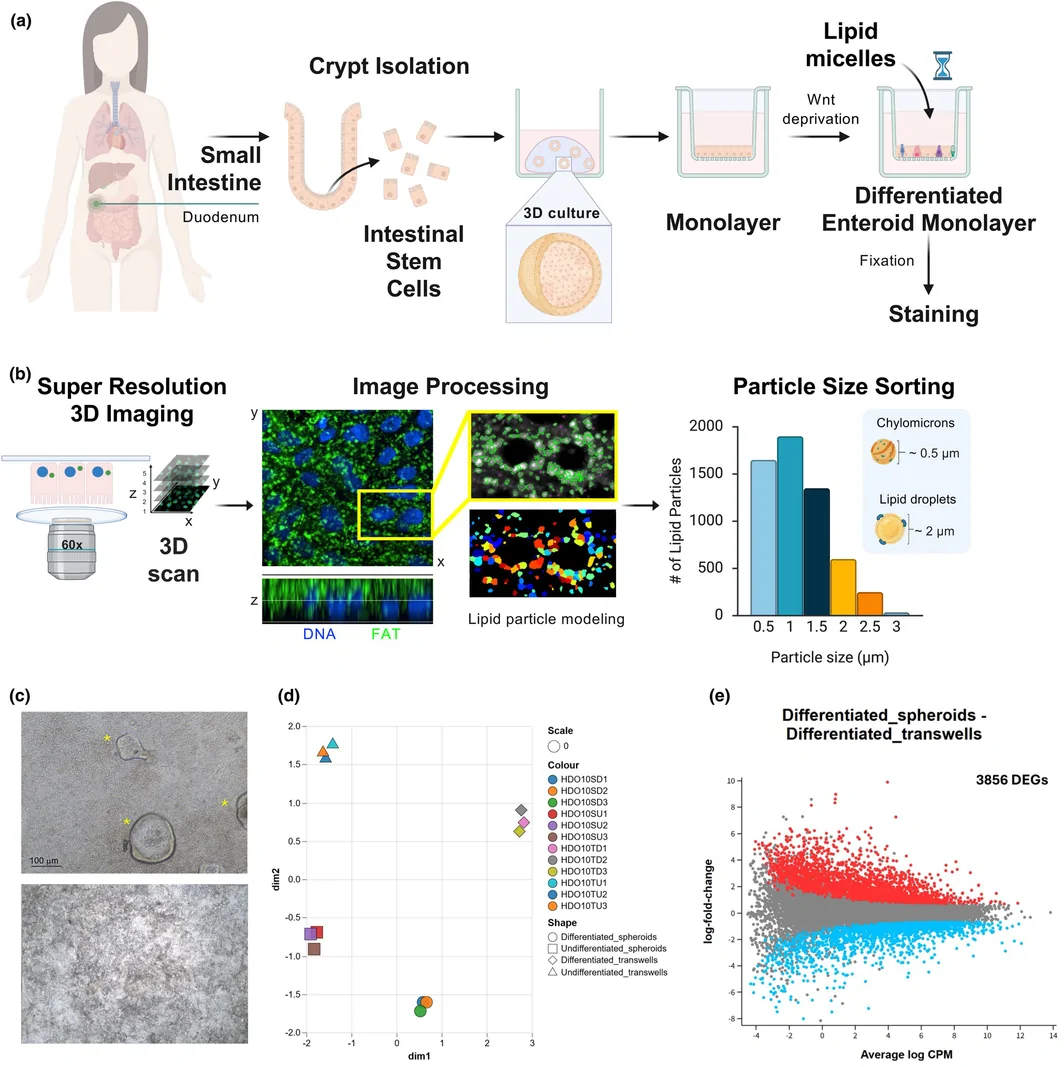
Generation and functional analysis of human‐derived intestinal organoids. (**a**) Human intestinal organoids are established from primary tissue, expanded in 3D culture, and dissociated to generate polarized epithelial monolayers on collagen‐coated Transwell inserts. Differentiation is induced by Wnt deprivation to promote enterocyte maturation. Monolayers are exposed to lipid micelles to stimulate postprandial fatty acid uptake and lipoprotein assembly, then fixed and stained for neutral lipids and lipid particle markers. (**b**) Super‐resolution spinning‐disk confocal microscopy is performed to acquire full‐thickness z‐stacks spanning the apicobasal axis. Image stacks are deconvolved and processed for background correction prior to segmentation. Lipid‐containing structures are identified using intensity‐ and morphology‐based thresholding and reconstructed as rendered objects. Particle size distributions are stratified aiming to distinguish cytosolic lipid droplets from chylomicron‐sized particles. Quantitative analyses are performed to compare lipid particle abundance and size profiles across experimental conditions. (**c–e**) Comparative studies of enteroids derived from a 5‐day old female donor in differentiated versus undifferentiated in 3D suspension culture or enteroid‐derived Transwells™ monolayers. (**c**) Cell morphology of undifferentiated (top) and differentiated (bottom) Transwells™ cultures. Note the presence of some intact spheroids (*) in undifferentiated culture. (**d**) PCA plot segregating cultures based on RNAseq analysis of the four tested conditions. (**e**) Volcano plot of RNAseq data derived from enteroids differentiated in 3D suspension versus Transwell™ cultures.

### MINeD

Dietary minerals, including metals (Cu, Zn, Ca, Mn, Fe) are essential nutrients for many physiological functions. However, the transport proteins critical for nutrient secretion during lactation and intestinal absorption are poorly defined. The TEC on Minerals In Nutrition and Development (MINeD) at the Johns Hopkins University is building the infrastructure and fundamental discovery pipeline to understand the physiology of human metal transport and nutrient uptake in the developing gut and adapting maternal intestine and breast. MINeD is defining the human transporter proteome and metallome in lactating mammary epithelium and how each relates to transport of nutrients into milk using human *in vitro* organoid models of lactating mammary glands derived from breast tissue, breast milk and human induced pluripotent stem cells. Complementary studies using adult and pediatric duodenal and jejunal enteroids are being used to determine the role of metal transporters in intestinal adaptation and nutrient absorption to support child development, maternal pregnancy and lactation (**Figures** **3**, **4**).

**Figure 4:**
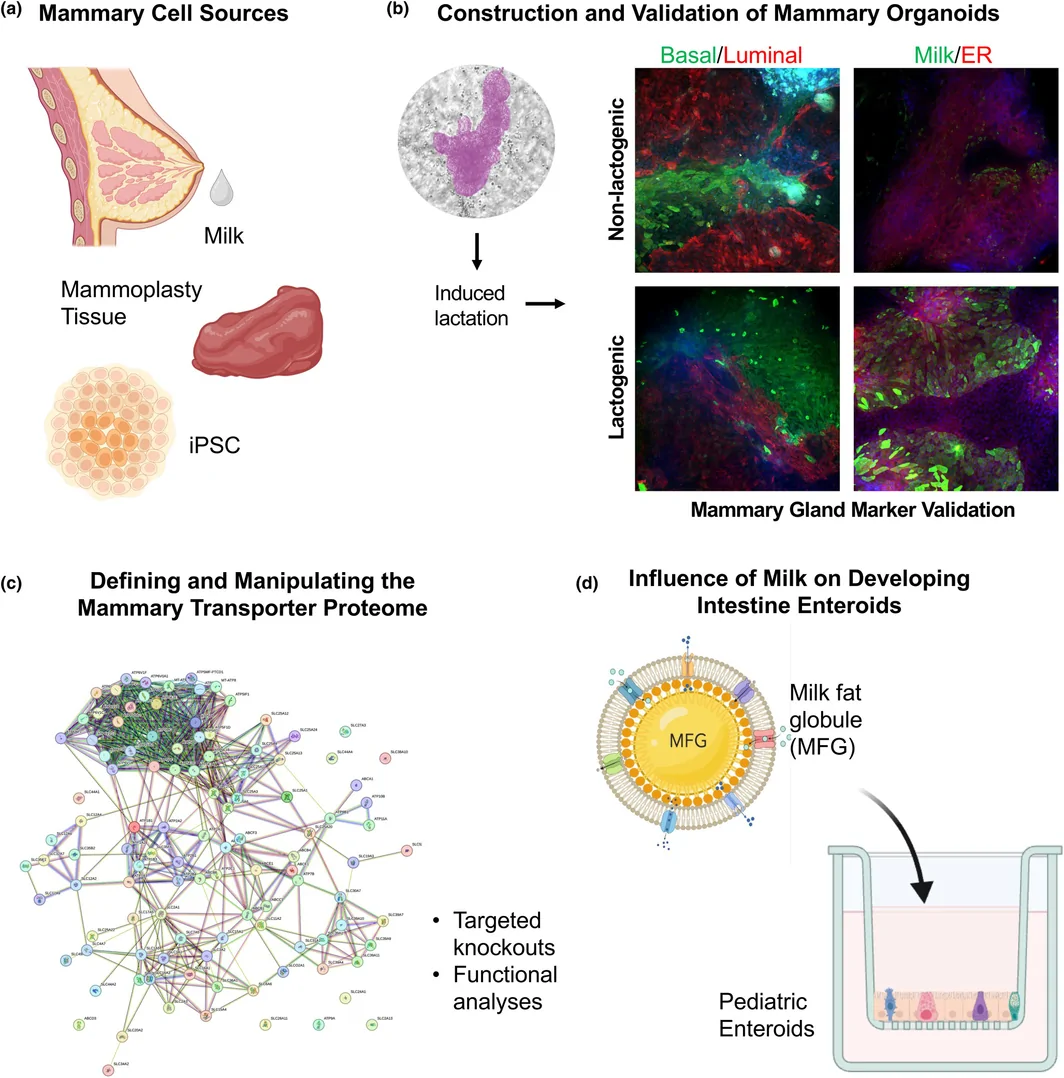
Schematic for identification and functional analysis of transporters in lactating mammary gland. (**a**) Human milk, mammary tissue obtained from healthy mammoplasty donors and human induced pluripotent cells have been used to (**b**) generate mammary organoids. Following induction of lactation by prolactin and validation using appropriate markers by immunofluorescence microscopy, the mammary transportome (**c**) was identified using membrane proteomics. Selected candidates will be subjected to knockdown and functional analysis. (**d**) Separately, uptake of fat from the human milk fat globules by pediatric enteroids will be analyzed as depicted in **Figure** **3** **a,b**.

### UCSF_TEC_

By mediating entry and exit into the brain, transporters in the polarized endothelial cells of the BBB play essential roles in homeostatic mechanisms of the central nervous system. The goal of the TEC at the University of California San Francisco (UCSF_TEC_) is to elucidate the ligand specificity, protein structure and cellular localization of SLC and ABC transporters in the human BBB. Using global proteomic methods, UCSF_TEC_ identified 30 highly expressed human BBB transporters revealing age‐dependent changes in their expression levels (**Figure** **1** **d**).^25^ However, a major gap exists in understanding the localization and specificities of these transporters in the BBB, which includes several orphan transporters. UCSF_TEC_ has prioritized four GABA transporters in the BBB, namely GAT‐1/*SLC6A1*, GAT‐3/*SLC6A11*, BGT‐1/*SLC6A12*, and GAT‐2/*SLC6A13*, by developing cell lines overexpressing each of these transporters. Imaging, flux, uptake studies and *in silico* docking are being performed on these transporters as well as several orphan transporters in the BBB. Finally, to develop a molecular understanding of transport mechanism, cryo electron microscopy (CryoEM) is being used, along with new techniques, to determine the structure of selected BBB transporters without available structures. This includes elucidating the structure of ABCD3, and resolving its mechanism for transport of substrates including branched‐chain fatty acids, very long‐chain fatty acids, bile salt intermediates, and dicarboxylic acids – this was accomplished from the structure with phytanoyl Co‐A (**Figure** **5**).^26^ This knowledge is critical to understanding nutrient transport of fatty acids, which plays a role in normal neonatal development. The effects of mutations in ABCD3 manifest during the perinatal period as defects in congenital bile acid synthesis and certain forms of Zellweger syndrome.

**Figure 5:**
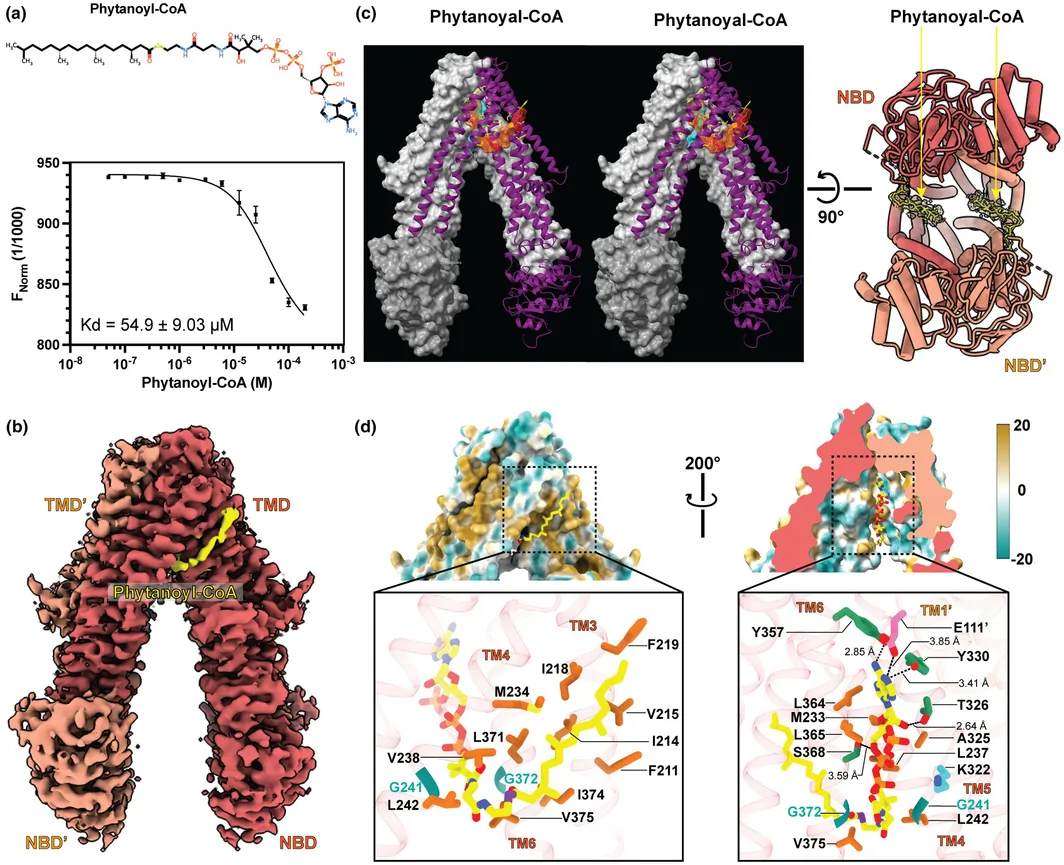
Structure of human ABCD3 bound to phytanoyl‐CoA using cryo electron microscopy. ABCD3 is the third most highly represented ABC transporter identified in the UCSF TEC project (**a**) The binding affinity of phytanoyl‐CoA for human ABCD3 was determined using MST to be 55 ± 9 μM (*n* = 3) (**b**) The CryoEM density map shows phytanoyl‐CoA bound to ABCD3. Density corresponds to phytanoyl‐CoA (yellow) protomer A (dark orange) and protomer B (light orange). (**c**) Crossed eyed stereo figure of phytanoyl coA bound to ABCD3. The front monomer is in purple cartoon, the rear monomer is in gray surface, with the NBD in dark gray surface, substrate in cyan. Phytanoyl CoA is in orange surface over the stick structure for the near monomer, in molecular surface for the back monomer. Side changes that interact with the near substrate are coloured in yellow stick representation. Right, view from the bottom. (**d**) Hydrophobicity surface view of ABCD3 around the box region shown in (**c**) showing the phytanoyl‐CoA binding site. The lipid tail of phytanoyl‐CoA outside the transmembrane helix cavity shown in upper left panel with insight of hydrophobic interaction network in lower left panel. Sectioned view of the ABCD3 substrate binding pocket with phytanoyl‐CoA bound (upper right panel) highlighting the interactions (lower right). Phytanoyl‐CoA (yellow), hydrophobic (orange), polar (green), negatively charged (pink), and positively charged (cyan); color by atom: oxygen (red), nitrogen (blue), and sulfur (violet).

## RELATIONSHIP OF THE TEN TO ADDITIONAL TRANSPORTER CONSORTIA

Over the past three decades, there have been several transporter‐focused working groups and consortia including the International Transporter Consortium, a collaboration of scientists from academia, industry, and the Food and Drug Administration^27^ with keen focus on transporters important in drug development.^1^, ^28^ The TEN leverages numerous scientists from the Consortium to investigate a wider array of SLC and ABC transporters involved in inherited genetic disorders, ontogeny, and disposition of broad chemical classes including nutrients, toxicants, and supplements. More recently, in 2020, the RESOLUTE Consortium was established as a private‐public venture to explore SLC transporters as novel drug targets.^29^ This collaboration has yielded a wide array of publicly available reagents, datasets, and searchable dashboards,^30^, ^31^ many of which are being leveraged by the TEN.

## NEEDS OF THE TRANSPORTER COMMUNITY

During registration for the 2025 meeting hosted by the TEN, participants (*N* = 72) provided open‐ended responses about the most urgent needs or challenges in transporter research. These responses were curated into key areas, and then real‐time polling was performed (*N* = 150 participants) during the beginning of the TEN meeting to rank challenges. Below and in **Table** **2**, we highlight how the TEN is working to address the majority of these needs, particularly as they relate to pregnancy and pediatric development and across organ systems with UWTEC and InTEC collaborating on placenta‐related projects and UWTEC and MINeD partnering to study intestinal transporter ontogeny.

**Table 2:** Urgent Needs and Challenges in Membrane Transporter Research Ranked Highest to Lowest and Mapped to Research Priorities in the TEN^a^

Needs and Challenges	InTEC	UWTEC	UCSF_TEC_	MINeD
Development of Advanced *In Vitro* Models	X	X	X	X
Sustainable Funding and Support			X	X
Better Probes, Assays, and Screening Tools	X	X	X	X
Transporter Function in Pregnancy and Lactation	X	X		X
Transporter Interplay and Localization	X	X	X	X
Mechanistic and Functional Coupling Studies	X	X	X	X
Integration with Multi‐Omics Approaches		X	X	
Transporter Regulation Mechanisms	X	X	X	X
*In Vitro‐In Vivo* Translation and IVIVE		X		
Precision Medicine and Genetic Variability	X			X
Transporter Function in Nontraditional and Pathological Tissues	X	X	X	X
Machine Learning and AI Applications	X		X	
Deorphaning of Transporters	X	X	X	X
Quantitative Systems Pharmacology and Modeling	X	X	X	
Improved Structural Characterization			X	
FDA Guidance and Regulatory Tools		X		
Data Standards and FAIR (Findable, Accessible, Interoperable, and Reusable) Practices	X	X		
Public and Clinical Communication			X	X

^a^ Seventy‐two registrants to a public meeting of the TEN provided open‐ended responses which were summarized into key areas. The two‐day meeting was held on Zoom in April 2025. With 150 participants from the academic, pharmaceutical industry, and government sectors, real‐time polling was performed to rank needs and challenges from highest to lowest. Two of the identified areas, development of novel drug delivery systems and isolation and enrichment of transporters, are not high priority areas for the TEN.

## LEVERAGING NOVEL APPROACHES TO ADDRESS TRANSPORTER COMMUNITY NEEDS

To begin to address the needs of the broader transporter community, the TEN is implementing a wide array of in silico, in vitro, in vivo, and translational approaches.

### Deorphaning transporters with metabolomics

About 30% of proteins in the SLC superfamily are designated as orphan transporters. One of the most powerful methods for deorphaning transporters is through metabolomics, notably, metabolomic‐genome wide association studies (mGWAS) and cellular metabolomic studies.

For mGWAS, samples (e.g., blood, cerebrospinal fluid) from human volunteers are profiled using liquid chromatography‐mass spectrometry methods (LC–MS), and genetic variants are identified from the DNA in the samples using genome wide technologies.^32^ The metabolites that associate with genetic variants in orphan transporters are candidate substrates for the transporters. Using this method, UCSF_TEC_ deorphaned, SLC22A24.^33^ Genetic variants in the transporter were highly associated with etiocholanolone glucuronide and androsterone glucuronide. Further studies were conducted in which the substrate specificities of the transporters were characterized, and transporters were localized to tissues.

In cellular metabolomic studies, individual transporters are overexpressed in cell lines and are exposed to fetal bovine calf‐serum, human serum or other biological samples. After exposure, the abundance of each metabolite is compared statistically to identify significant differences between control and transporter‐expressing cell lines. This method has been applied by UCSF_TEC_ to deorphan FLIPT/*SLC22A15* ^34^ and to discover additional substrates for the organic cation transporter, OCT1/*SLC22A1*.^35^ SLC22A15 was determined to have activity for ergothioneine, a powerful antioxidant that is found at high levels in the brain. Other orphan transporters with no known ligands include SLC22A17, SLC22A23, SV2A, and SV2B. One of the goals of UCSF_TEC_ studies is to apply metabolomic methods to identify potential substrates of orphan transporters.

### Transporter proteomics

Transporter expression in mammary, placenta, and fetal organs has been primarily quantified at the mRNA level. However, mRNA expression often fails to correlate with protein expression and activity.^36^, ^37^ The UWTEC is applying global and targeted proteomics to identify and quantify transporter abundance in the developing placenta and gastrointestinal tract.^8^, ^9^, ^21^ Given the large numbers of SLC and ABC transporters in humans, the UWTEC is initially using a data‐independent acquisition workflow that provides an unbiased approach to determine abundances of a broad range of nutrient and xenobiotic transporters.^38^, ^39^ Highly expressed transporters with unknown function and transporters with gestational age‐associated changes in abundance will be further characterized with targeted proteomics to confirm concentrations in the placenta and developing gastrointestinal tract. Similarly, UCSF_TEC_ has applied global proteomic methods to identify SLC and ABC transporters in the human BBB, discovering developmental changes in many transporters.^25^ MINeD is using proteomics to identify transporters in mammary tissue as well as the milk fat globule, a membrane‐bound component of milk secreted by lactocytes within the alveoli of the lactating mammary gland. By comparison, InTEC is relying entirely on targeted proteomics known as quantitative targeted absolute proteomics (QTAP) to quantify ~60 transporters using membrane proteins extracted by differential detergent fractionation.^40^, ^41^, ^42^, ^43^ The transporter proteome is being defined in healthy and diseased human placental tissues as well as commonly used placental cell lines and explants and extracellular vesicles derived from placental perfusion media studies. The complementary approach of using both bottom‐up and top‐down proteomics within the TEN will allow comprehensive profiling of the perinatal transporter proteome.

### Perfused placenta/ex vivo models

The perfused human placenta is an excellent *ex vivo* model to investigate the mechanism and extent of placental transfer of nutrients and xenobiotics. This model simulates maternal and fetal perfusion through human cotyledons and retains a functional placental barrier.^44^ It avoids the deficiencies of other models which are either animal‐based (*in vitro* or *in vivo*) or based on placental cell lines.^45^, ^46^ However, because it lacks the fetal compartment, the perfused placenta cannot always predict the extent of *in utero* exposure to nutrients or xenobiotics when they are significantly metabolized by the fetal organs. In addition, this model requires special expertise and equipment not available in many laboratories and is often limited to the perfusion of term placenta. Nevertheless, the perfused placenta model has been successfully used to predict the transfer of xenobiotics from the maternal intervillous space to the fetal vasculature.^47^, ^48^, ^49^, ^50^ The UWTEC will utilize this model to assess the mechanisms and extent of transport of substrates by the transporters identified in the term human placenta by transcriptomics and/or proteomics.

### Organoids and Mammospheres

To understand the postnatal ontogeny of transporter expression, UWTEC is building a library of intestinal donor tissues between 0 and 6 years old. In addition to proteomic profiling of native duodenal/jejunal/ileal tissues, organoid cell lines are being derived by propagating crypt stem cells that express the leucine‐rich G repeat‐containing protein‐coupled receptor 5 (LGR5) marker.^51^ These “spheroids” can replicate *in vitro* ad infinitum in their undifferentiated state and can be induced to differentiate by withdrawal of growth factors. Following differentiation, these “enteroids” express drug metabolizing enzymes and transporters associated with endogenous human intestinal tissues.^52^ Ontogenic biology and function of intestinal transporters are being profiled using postnatal enteroid‐derived intestinal monolayers cultured in a Transwell™ system, an industry standard for modeling intestinal permeability and facilitated transport.^53^ Unlike Caco‐2 monolayers, enteroid‐derived monolayers also express a full complement of drug metabolizing enzymes as well as ABC/SLC transporters allowing modeling of more complex drug transport and metabolism phenomena.^51^, ^54^ Compared to enteroid MPS culture conditions completely deprived of Wnt3a, which drives terminal differentiation and limited length of viability, the enteroid MPS is viable for at least 6 weeks and retains multiple cell types found in the small intestine based on deconvolution of RNA sequencing data.^54^, ^55^

In parallel, the MINeD team is defining the human ‘transportome’ in lactation‐induced mammary organoids derived from healthy donor breast tissue and cross‐checked by proteomic analysis of milk fat globule membranes in donor derived milk. Using gene‐editing technology, investigators at MINeD are tracking endogenous expression of metal transporters and establishing functional links to fat uptake and accumulation in intestinal models.

### Microphysiological Systems (MPS)

Researchers at InTEC are at the forefront of leveraging MPS technology to address these challenges and investigate the biology of placental transport in human‐relevant conditions. Recently, the center has developed a microengineered platform to culture primary human placental cells in 3D and allow them to self‐organize into a multilayered tissue construct composed of a syncytialized trophoblast layer and an underlying vascularized stroma that can be perfused in a controlled manner (**Figure** **2**). Designed to mimic the placental barrier that separates maternal blood in the intervillous space from fetal circulation in the chorionic villi, this MPS model expresses key transporter proteins, such as GLUT1/*SLC2A1*, BCRP/*ABCG2*, and OATP2B1/*SLCO2B1*, found in the human placenta and provides an *in vitro* platform to simulate and interrogate molecular exchange between maternal and fetal blood. This placenta‐chip was recently used to evaluate the transporter‐mediated accumulation of the toxic metal cadmium, but also the differential toxicity that cadmium exerts on maternal trophoblasts versus fetal vasculature and the role of Hofbauer cells and neutrophils in this toxicity.^56^ Taking advantage of design flexibility and precise control of experimental variables afforded by our MPS platform, InTEC is also studying how organ‐specific environmental cues such as hemodynamic force and intercellular signaling affect the expression and functional capacity of key placental transporters.

### Machine learning (ML) and artificial intelligence

Over the past decades, a large number of chemicals have been tested for their interaction with various transporters, resulting in a vast amount of transporter binding data.^57^, ^58^ ML, a key component of artificial intelligence, has proven particularly well‐suited for modeling transporter substrates and inhibitors due to its ability to capture complex relationships between diverse chemical structures and bioactivities.^59^ P‐gp/*ABCB1* was one of the first ABC transporters to be discovered, leading to a substantial accumulation of data and early ML‐based models on its inhibitors and substrates.^60^, ^61^ In a recent study, model training was performed using 8777 compounds for P‐gp inhibition and substrate binding using the combination of ML and molecular dynamics.^62^ In contrast, ML models for SLC transporters have been based on much smaller datasets, as many SLC transporters remain poorly characterized.^63^ Since modern ML methods, particularly deep neural networks (DNNs), require large datasets to demonstrate their full potential, several major efforts have been initiated to generate, curate, and share comprehensive transporter data. Public chemical data portals such as PubChem and ChEMBL, along with transporter‐specific databases like Metrabase,^64^ TP‐Search,^65^ the UCSF‐FDA TransPortal,^66^ the Transporter Classification Database,^67^ and VARIDT,^68^ provide structural, functional, genomic, and mRNA data available for advanced ML approaches.^69^, ^70^ A popular strategy in these studies is hybrid modeling, which integrates multiple modeling techniques to overcome the limitations of individual approaches and enhance prediction performance.^71^, ^72^ Moreover, a growing trend in the field is the development of user‐friendly, web‐based prediction platforms to make transporter binding models accessible to researchers around the world.^69^, ^73^ The gathering of transporter binding and inhibition data and the availability of ML‐based transporter models has advanced the modeling studies of other drug properties and activities, especially transporter‐related pharmacokinetic modeling.^74^, ^75^, ^76^

### PBPK modeling

Although, as indicated above, the perfused placenta can be used to predict fetal exposure to nutrients or xenobiotics, it may not reflect exposure to these compounds at earlier stages of gestation or predict maternal exposure. These predictions can be made using PBPK modeling. Such m‐f PBPK models incorporate all the changes in maternal physiological parameters throughout pregnancy, including blood flow to the maternal liver, kidneys, and placenta.^15^ In contrast, most fetal physiological parameters are available from about gestational week 15 to term, allowing prediction of fetal exposure to nutrients or xenobiotics during this time window.^16^ Important amongst these parameters is the abundance (or activity) of transporters and metabolizing enzymes in various maternal and fetal organs that process the nutrient and xenobiotic of interest. Although placental transport and metabolism rates or clearances can be obtained from the perfused placenta studies, such studies are relatively low throughput, not only due to technical complexity but also reliance on fresh (viable and not coagulated) placenta donations. A better and higher throughput approach is to conduct *in vitro* metabolism studies using subcellular fractions of the placenta and fetal tissue to determine the rate of metabolism of the compound of interest. Then, transport studies with cell lines that express the relevant transporters can be conducted. The transporters are usually overexpressed and their abundance in cell lines does not reflect the concentrations in human tissues. To account for this difference, the abundance of the transporter can be quantified using QTAP. M‐f PBPK models, populated with these data, have been successfully used to predict not only maternal exposure, but also fetal exposure to xenobiotics.^45^, ^77^, ^78^, ^79^, ^80^, ^81^, ^82^

### Extracellular vesicles and liquid biopsies

A key challenge in the placenta field is access to healthy human placentas prior to full‐term delivery. This limitation has initiated interest in alternative, minimally invasive approaches to study placenta biology. Extracellular vesicles (EVs), shed from placenta cells into the maternal circulation, express surface proteins or carry cargo, such as proteins, lipids, DNA, and various forms of RNA.^83^, ^84^ Plasma and serum‐derived EVs are already being investigated as disease biomarkers and drug delivery systems, and mediators of immune modulation.^85^, ^86^ More recently, EVs have been evaluated for quantifying hepatic drug metabolizing enzymes and transporters to study drug exposure variability.^87^, ^88^, ^89^ One of these studies has shown the impact of pregnancy on CYP3A4, CYP2D6, and OATP1B3/*SLCO1B3* expression in serum‐derived hepatic EVs.^87^ Plasma−/serum‐derived placental EVs can also be enriched with the surface marker placental alkaline phosphatase using different techniques, such as affinity‐based capture or multiplexed nanoscale high resolution flow cytometry/sorting approach.^90^, ^91^ The number of plasma‐derived EVs and the expression of placental alkaline phosphatase have been shown to increase with gestational age.^90^ InTEC aims to evaluate longitudinal changes in SLC/ABC transporters localized on the apical surface of syncytiotrophoblasts throughout gestation. Though challenges remain in standardization and clinical validation, the ability to use placental EVs as a surrogate measure of placenta biochemical status holds great promise for evaluating transporter changes throughout pregnancy and with disease.

### Birth cohorts/clinical studies

Longitudinal birth cohorts are a rich source of biospecimens and data that provide insight into how early events in pregnancy can shape the growth and developmental trajectories of offspring. By design, they offer a temporal sequence between exposure, whether nutritional, social, or environmental, and later outcomes to cognition, metabolism, and disease. In recent years, birth cohorts have been used to study how non‐synonymous polymorphisms in xenobiotic transporters such as BCRP/*ABCG2*, OATP2B1/*SLCO2B1*, and OAT4/*SLC22A11* can influence fetoplacental outcomes following exposure to environmental metals such as cadmium^92^ and pesticides^93^ as well as dietary contaminants such as mycotoxins.^40^ For example, *in utero* exposure to the estrogenic fungal toxin zearalenone was inversely associated with birth weight most notably in infants with the Q141K polymorphism in *ABCG2/*BCRP,^40^ consistent with animal studies demonstrating that BCRP restricts placental and fetal accumulation of this widespread food contaminant.^10^ By using targeted proteomics, enrichment of placental transporters such as BCRP according to genotypes can be quantified.^40^ InTEC is partnering with the Understanding Pregnancy Signals and Infant Development (UPSIDE) birth cohort.^94^ UPSIDE is a prospective pregnancy and birth cohort out of the University of Rochester Medical Center that was designed to study how maternal stress, psychological factors, and environmental exposures during gestation influence placental function, neurodevelopment, and overall child health. InTEC is leveraging the rich biospecimens and extant data in UPSIDE to study transporter genetics and proteomics and relationships to dietary and environmental exposures that influence metabolic and neurological development into adolescence. This cohort will provide great insight into potential determinants of inter‐individual enrichment of SLC and ABC transporters in human placentas.

Additional resources for accessing biospecimens and human datasets for further transporter research include federal resources such as NICHD Data and Specimen Hub (DASH)^95^ and the Environmental Influences on Child Health Outcomes (ECHO)^96^ as well as biorepositories that include the Global Pregnancy Collaboration^97^ and the PeriBank.^98^ The Collaborative Online Pediatric and Perinatal Repository (COPPER) maintains a listing of biobanks related to pregnancy and pediatrics.^99^

## FUNDING

This work was supported by the NIH Office of the Director, National Institute on Drug Abuse, and *Eunice Kennedy Shriver* National Institute of Child Health and Human Development [Grants UC2HD113039, UC2HD113474, UC2HD113036, and UC2HD113041].

## CONFLICT OF INTEREST

The authors declared no competing interests for this work.

## DISCLAIMER

The views expressed in this publication are those of the authors and do not necessarily reflect the official policy or position of the Defense Health Agency, Department of War, nor the U.S. Government. The study protocol was approved by respective Institutional Review Boards in compliance with all applicable Federal regulations governing the protection of human subjects.

## SUMMARY AND FUTURE OUTLOOK

Through collaborative research and advancement of experimental and computational approaches, the TEN is actively identifying novel functions and regulators of nutrient, supplement, drug, and toxicant transporters important for prenatal and postnatal development. As datasets, models, and resources are validated, they are being actively disseminated to public databases to support the broader transporter community. Lastly, the TEN is committed to training the next generation of transporter scientists aimed at improving pediatric pharmacology and clinical outcomes.
